# Genome size variation and evolution in allotetraploid *Arabidopsis kamchatica* and its parents, *Arabidopsis lyrata* and *Arabidopsis halleri*

**DOI:** 10.1093/aobpla/plu025

**Published:** 2014-05-26

**Authors:** Diana E. Wolf, Janette A. Steets, Gary J. Houliston, Naoki Takebayashi

**Affiliations:** 1 Department of Biology and Wildlife, Institute of Arctic Biology, University of Alaska Fairbanks, 311 Irving I, Fairbanks, AK 99775-7000, USA; 2 Present Address: Department of Botany, Oklahoma State University, 301 Physical Sciences, Stillwater, OK 74078-3013, USA; 3 Present Address: Landcare Research, Gerald St, Lincoln 7608, New Zealand

**Keywords:** Allotetraploid, *Arabidopsis halleri* ssp. *gemmifera*, *Arabidopsis kamchatica*, *Arabidopsis lyrata*, C-value, 2C DNA content, flow cytometry, genome size, genome size variation

## Abstract

Polyploidization and subsequent changes in genome size are fundamental processes in evolution and diversification. Little is currently known about the extent of genome size variation within taxa and the evolutionary forces acting on this variation. *Arabidopsis kamchatica* has been reported to contain both diploid and tetraploid individuals. The aim of this study was to determine the genome size of *A. kamchatica*, whether there is variation in ploidy and/or genome size in *A. kamchatica* and to study how genome size has evolved*.* We used propidium iodide flow cytometry to measure 2C DNA content of 73 plants from 25 geographically diverse populations of the putative allotetraploid *A. kamchatica* and its parents, *Arabidopsis lyrata* and *Arabidopsis halleri*. All *A. kamchatica* plants appear to be tetraploids. The mean 2C DNA content of *A. kamchatica* was 1.034 pg (1011 Mbp), which is slightly smaller than the sum of its diploid parents (*A. lyrata*: 0.502 pg; *A. halleri*: 0.571 pg). *Arabidopsis kamchatica* appears to have lost ∼37.594 Mbp (3.6 %) of DNA from its 2C genome. Tetraploid *A. lyrata* from Germany and Austria appears to have lost ∼70.366 Mbp (7.2 %) of DNA from the 2C genome, possibly due to hybridization with *A. arenosa*, which has a smaller genome than *A. lyrata*. We did find genome size differences among *A. kamchatica* populations, which varied up to 7 %. *Arabidopsis kamchatica* ssp. *kawasakiana* from Japan appears to have a slightly larger genome than *A. kamchatica* ssp. *kamchatica* from North America, perhaps due to multiple allopolyploid origins or hybridization with *A. halleri*. However, the among-population coefficient of variation in 2C DNA content is lower in *A. kamchatica* than in other *Arabidopsis* taxa. Due to its close relationship to *A. thaliana*, *A. kamchatica* has the potential to be very useful in the study of polyploidy and genome evolution.

## Abstract (precis)

Genome doubling and changes in genome size are fundamental evolutionary processes. *Arabidopsis kamchatica* has been reported to contain both diploid and tetraploid individuals (2 or 4 copies of every chromosome). We did find genome size differences among populations, and among populations genome size varied 7%. However, all sampled *A. kamchatica* plants from a wide geographic range were tetraploids. This level of intraspecific genome size variation in *A. kamchatica* is lower than in other *Arabidopsis* taxa. Due to its close relationship to *A. thaliana*, *A. kamchatica* has the potential to be very useful in the study of polyploidy and genome evolution.

## Introduction

Polyploidy is one of the most important forces influencing plant diversification. Polyploidy was likely involved in 15 % of all recent angiosperm speciation events (Wood *et al.* 2009) and ancient polyploidy is apparent in all plant genomes sequenced to date (Jiao *et al.* 2011). Similarly, the majority of cultivated crops have undergone polyploidization during domestication (Otto and Whitton 2000). Polyploidy influences the ecology and physiology of plants by generating novel phenotypes that may influence mating system, habitat and geographical distribution (Levin 2002). It can have major genetic and genomic effects, such as altering chromosome segregation, masking deleterious mutations, influencing levels of genetic diversity, changing gene expression, causing rearrangements, gene loss and epigenetic changes, rewiring genetic networks, and altering rates of adaptation (Levin 2002; Adams and Wendel 2005; Chen 2007; De Smet and Van de Peer 2012; Madlung 2013). Ploidy variation has the potential to promote the origin of new species, but ploidy variation within species (or species complexes) may also be an important source of genetic and phenotypic variation (Thompson and Lumaret 1992). Thus, plant biodiversity cannot be understood without understanding the processes of polyploid evolution (Lutz 1907; Stebbins 1950; Grant 1981; Madlung 2013).

Polyploids are thought to experience high levels of genomic instability and undergo massive genetic and epigenetic changes within the first few generations after formation (Chen 2007). It is likely that a great deal of genomic and phenotypic diversity is generated and the majority of early generation polyploids are unable to survive in nature. However, if one or a few stable genotypes arise that happen to reconcile genomic incompatibilities, are vigorous and are well suited to survival in the prevailing habitat, polyploids can persist (Chen 2007; Madlung *et al.* 2012). After this rapid ‘genomic revolution’, it is likely that a slow process of diploidization begins, where gene duplicates may be silenced, lost or evolve new functions (Wolfe 2001). It is thought that nearly all angiosperms have experienced at least one polyploidy event in their evolutionary history (Wolfe 2001). However, due to extensive mutation, gene loss and rearrangements, these diploidized paleopolyploids, such as *Arabidopsis thaliana*, have only recently been recognized as whole-genome sequences became available for detailed analysis (Vision *et al.* 2000). Both the rapid genomic revolution and gradual process of diploidization are likely to result in variation and evolution in genome size as DNA is deleted, duplicated and rearranged, and variants are subject to genetic drift and selection.

Polyploidy can arise from the duplication of genomes within a single species (autopolyploidy) or through hybridization between two species, accompanied by chromosome doubling (allopolyploidy) (Levin 2002). Either allopolyploidy or autopolyploidy may arise via a single polyploidization event, like in *Arabidopsis suecica* (Säll *et al.* 2003; Jakobsson *et al.* 2006), or may have multiple origins (Soltis and Soltis 1999), as has been suggested for *A. kamchatica* (Shimizu-Inatsugi *et al.* 2009). Further, variation in ploidy level is frequently found within species both within and among populations (Schmuths *et al.* 2004; Marhold *et al.* 2010), and gene flow between ploidy levels is known to occur, either via a triploid bridge or through recurrent formation of unreduced gametes by diploids (Levin 2002; Husband 2004; Henry *et al.* 2005, 2009; Jørgensen *et al.* 2011). This gene flow from diploids to polyploids is likely an important source of genetic variation in polyploids (Jørgensen *et al.* 2011).

*Arabidopsis kamchatica* is an allotetraploid plant produced through hybridization through two closely related diploid taxa, *Arabidopsis lyrata* ssp. *petraea* and *Arabidopsis halleri* ssp. *gemmifera* (Shimizu *et al.* 2005; Shimizu-Inatsugi *et al.* 2009). *Arabidopsis kamchatica* has an amphi-Beringian distribution, and the pattern of genetic diversity suggests that it migrated northward out of Japan (or near Japan) to eastern Russia, across the Bering land bridge into Alaska, and down the west coast of Canada (Shimizu-Inatsugi *et al.* 2009). It has been suggested that *A. kamchatica* may have multiple origins through independent hybridization and polyploidization events (Shimizu-Inatsugi *et al.* 2009), and/or that it may hybridize with its diploid parental taxa (Shimizu-Inatsugi *et al.* 2009; Wang *et al.* 2010). Both of these processes have the potential to give rise to genome size variation. Further, *A. kamchatica* has been suggested to contain both diploid and tetraploid individuals (Dawe and Murray 1981; Wang *et al.* 2010). Because *A. kamchatica* is a close relative of the model plant, *A. thaliana*, a treasure trove of molecular research is easily applied to this organism, and development of *A. kamchatica* into a model system for the evolution of polyploidy has the potential to yield a great deal of insight into the evolution of polyploid genomes.

The goal of this study was to investigate genome size variation in *A. kamchatica* using flow cytometry. We characterized the nuclear DNA content of *A. kamchatica* and its putative parental species, *A. lyrata* and *A. halleri*, in a total of 25 populations from North America, Europe and Japan. We used the results to determine whether there is variation in ploidy and/or genome size in *A. kamchatica* and its parents, and to determine how genome size has evolved in polyploids relative to their diploid parents.

## Methods

### Plant material

**Figure 1.**
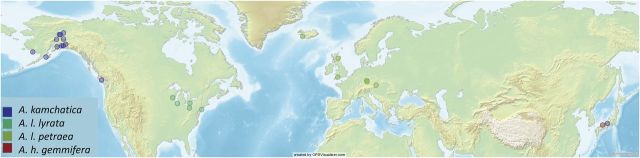
Map of collection localities of plants used for flow cytometry.

We estimated genome size from a total of 73 samples from *A. kamchatica* and its parental taxa *A. lyrata* (subspecies *A. l. lyrata* and *A. l. petraea*) and *A. halleri* ssp. *gemmifera* (Table 1, Fig. 1). All plants were germinated from seed and grown in the Institute for Arctic Biology Greenhouse at the University of Alaska Fairbanks. In populations with multiple samples, we sampled plants from different maternal families.

**Table 1.** Collection locations, collectors and mean (±1 SE) genome size of each population. ^1^Assumes *Glycine max* ‘Polanka’ 2C DNA content of 2.5 pg (Doležel *et al.* 1994; Doležel and Greilhuber 2010). ^2^Populations with different letters have significantly different means (*P* < 0.05) in post hoc comparisons among *A. kamchatica* populations with >2 individuals. ^3^Conversion from pg to Mbp assuming Mbp = pg × 978 (Doležel *et al.* 2003).

Taxon	Location	Latitude	Longitude	Collector/donor	Sample size	2C DNA content (pg)^1,2^	SE	Ploidy (2C)	2C genome size (Mbp)^3^
*A. h. gemmifera*	Japan	34.93	133.63	Fujita Corp.	9	0.571	0.0127	2*x*	558.35
*A. kamchatica*	USA, Alaska								
	Bear Creek	65.41355	−145.62545	C. Parker	1	1.013	NA	4*x*	990.43
	Chena River	64.82	−147.32	N.T., D.E.W.	4	1.023^AB^	0.0035	4*x*	1000.06
	Fairbanks	64.83333333	−147.7	C. Parker	1	1.025	NA	4*x*	1002.51
	Goodnews Bay	59.11666667	−161.583333	C. Parker	5	1.016^A^	0.0056	4*x*	994.06
	Grant Lagoon, Kodiak Island	57.37	−154.65	C. Parker	3	1.043^B^	0.0054	4*x*	1020.23
	Liberty Falls	61.62	−144.55	D.E.W.	1	1.039	NA	4*x*	1015.72
	Portage Glacier	60.79161667	−148.9021333	N.T., D.E.W.	3	1.039^AB^	0.0104	4*x*	1016.02
	Parks Highway	63.25	−149.25	N.T., D.E.W.	6	1.035^AB^	0.0035	4*x*	1012.30
	Rainbow Ridge	63.32	−145.64	N.T., D.E.W.	3	1.032^AB^	0.0053	4*x*	1009.16
	Shoup Bay	61.13	−146.59	N.T., D.E.W.	4	1.033^AB^	0.0050	4*x*	1010.30
	Thompson Pass	61.13	−145.73	N.T., D.E.W.	1	1.033	NA	4*x*	1010.20
	Canada, Vancouver Island								
	Strathcona Park	49.82915	−125.8728	J.A.S., D.E.W.	15	1.027^AB^	0.0032	4*x*	1004.24
	Japan, Honshu Island								
	Lake Biwa, Shinbo	35.44444444	136.05	H. Marui	5	1.083^C^	0.0027	4*x*	1059.29
*A. l. lyrata*	USA, Michigan, Grand Mere	42.01	−86.54	J.A.S.	1	0.525	NA	2*x*	513.27
	New York	40	−74	T. Mitchell-Olds	1	0.479	NA	2*x*	468.72
	Pennsylvania, Presque Isle	42.14	−80.11	J.A.S.	1	0.510	NA	2*x*	499.19
	Pennsylvania, Raccoon Creek	40.51	−80.34	J.A.S.	2	0.502	0.0097	2*x*	491.38
	Wisconsin	44	−89	T. Mitchell-Olds	1	0.498	NA	2*x*	486.70
*A. l. petraea*	England, Exeter	50.72	−3.53	T. Mitchell-Olds	2	0.477	0.0082	2*x*	466.68
	Germany, Plech	49.65	11.47	T. Mitchell-Olds	2	0.494	0.0034	2*x*	482.82
	Iceland, Reykjavik, Esja Mountain	64.2	−21.7	M. Schierup	2	0.526	0.0010	2*x*	513.96
	Scotland, Braemer	57.01	−3.4	R. Ennos	1	0.514	NA	2*x*	502.68
	Austria, Mödling	48.08	16.32	S. Ansell	1	0.922	NA	4*x*	901.93
	Germany, Dürn	49.27	11.6	T. Mitchell-Olds	1	0.941	NA	4*x*	920.06

### Ploidy determination

Chromosome counting is the traditional method for determining ploidy level of an organism; however, it is labour intensive and may be inaccurate in *Arabidopsis* species due to their very small chromosomes (ranging from 1.5 m to 2.8 µm in *A. thaliana*; Schweizer *et al.* 1987) and the high frequency of endopolyploidy (Galbraith *et al.* 1991; Melarango *et al.* 1993). Flow cytometry allows rapid analysis of thousands of nuclei per sample and high throughput of many samples (Kron *et al.* 2007). Therefore, we used flow cytometry to estimate genome size and infer DNA ploidy. Because flow cytometry reveals genome size rather than a count of chromosomes, ploidy must be verified by chromosome counts in at least a few samples. In our study, we included both diploid and tetraploid references from *Arabidopsis* locations where both flow cytometry and chromosome counts have previously been carried out (*A. kamchatica* from Japan, and *A. l. petraea* from Iceland and Austria; Table 1; Dart *et al.* 2004).

### Flow cytometry

Each *Arabidopsis* sample was co-chopped and run with soybean leaf, *Glycine max* ‘Polanka’, as an internal reference standard. The standard was grown from the same seed stock previously quantified (Doležel *et al.* 1994). Young leaves were collected from each *Arabidopsis* plant and kept on ice until processing, which occurred within 3 h of leaf collection. For each plant, three fresh leaves were placed in a plastic Petri dish with approximately half as much fresh leaf tissue from *G. max*. Leaf tissue was chopped in the presence of 0.5 mL of cold chopping buffer using a fresh stainless-steel razor blade. The chopping buffer was modified from Otto (1990) Buffer I by adding 0.5 % v/v of Triton X-100 rather than Tween 20. When the leaves were well chopped, we added an additional 0.5 mL of cold chopping buffer. The sample was then filtered through a 30-µm Partec CellTrics^®^ filter and centrifuged for 20 s at 3500 rpm. The supernatant was drawn off and 2 µL of RNase A was added to the pellet. The pellet was resuspended in 0.2 mL of propidium iodine staining buffer. The propidium iodine staining buffer (28.65 g of dibasic sodium phosphate, 190 mL of deionized water and 10 mL of propidium iodine stock, which consists of 5 mg of propidium iodine and 10 mL of deionized water) was modified from Otto (1990). Samples were stained in the dark for 40 min prior to performing flow cytometry.

**Figure 2.**
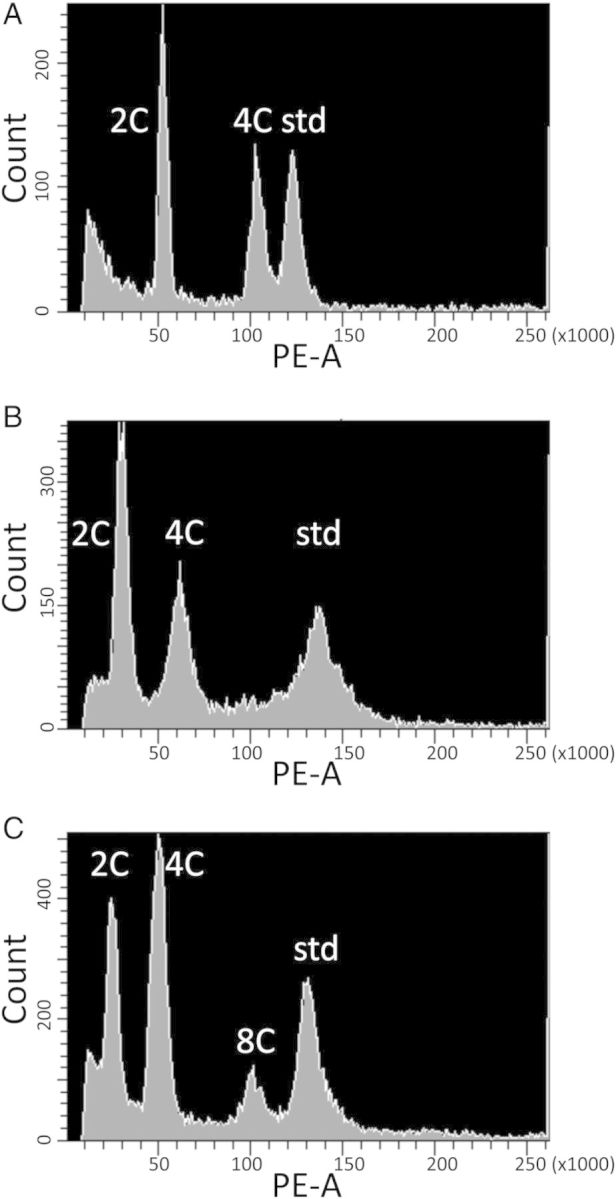
Fluorescence intensity histograms (PE-A) for (A) tetraploid *A. kamchatica* (2C = 4*x* = 32), (B) diploid *A. h. gemmifera* (2C = 2*x* = 16) and (C) diploid *A. lyrata* (2C = 2*x* = 16). *Arabidopsis* leaves show extensive endopolyploidy (Galbraith *et al.* 1991), and the 2C, 4C and 8C peaks are indicated, along with the soybean standard (std). The mean fluorescence of the smallest peak (2C) relative to the soybean peak was used to estimate 2C DNA content.

Flow cytometry was performed on a BD Biosciences FACSAria flow cytometer (BD Biosciences, San Jose, CA, USA) equipped with FACSDiva Software (BD Biosciences), using a Coherent Sapphire Solid State laser (488 nm) as the excitation source. Noise signals derived from subcellular debris were eliminated by gating. Samples were run until 5000 *Arabidopsis* nuclei were scored. Since propidium iodide was used to stain the nuclei, fluorescence was measured using the R-phycoerythrin (PE) detector, which uses the 576/26 nm bandpass filter. 2C DNA content was estimated from gated fluorescence histograms of PE area (Fig. 2). Due to endopolyploidy, the populations of plant nuclei typically gave multiple peaks of fluorescence, representing 2C, 4C and 8C nuclei (and sometimes even higher endopolyploid levels) (Galbraith *et al.* 1991; Melarango *et al.* 1993). The 2C DNA content of each sample was calculated using the smallest of the peaks, and comparing it to the *G. max* standard ((sample fluorescence/soybean fluorescence) × 2.5 pg; Doležel *et al.* 1994). All samples had a coefficient of variance (CV) for relative fluorescence among nuclei that was <10 %; however, only 48 % of samples had a CV ≤5 %, as recommended (Doležel *et al.* 2007). We believe that this is due in part to the very small *Arabidopsis* genome (Doležel *et al.* 2007), as the larger soybean standard peak had a mean CV of 3.32 %, and only 6.1 % of the samples had a CV >5 %. All soybean samples had a CV ≤5.7 %. To ensure that genome size measurements were repeatable, eight samples were repeated on different days. Differences between repeat measurements never exceeded 1.1 %, indicating that genome size measurements were highly repeatable (Doležel *et al.* 2007).

To determine whether the taxa differed in genome size, we used a linear mixed-effects model with species as the fixed effect, populations as the random effect and 2C DNA content (pg) as the dependent variable with the *lme4* package (Bates 2005), implemented in R. The hypothesis test of the species effect was conducted with 5000 iterations of the parametric bootstrap approach based on the likelihood ratio statistics, *D* = −2 × (log-likelihood ratio), of Faraway (2006). To determine which species differed from one another in 2C DNA content, we performed Tukey's multiple comparison tests with an R package, *multcomp* (Hothorn *et al.* 2008). To determine whether populations of *A. kamchatica* differed in 2C DNA content, a second one-way ANOVA was performed with population as the fixed effect and 2C DNA content (pg) as the dependent variable. For this analysis we restricted our dataset to include only the nine *A. kamchatica* populations for which we had at least three samples. The mean number of samples per population was 5.3. Tukey's multiple comparison tests were performed to determine which *A. kamchatica* populations significantly differed from one another in genome size. In order to test the additivity of the tetraploid genome size, we examined a contrast null hypothesis, where the 1Cx genome size (i.e. the haploid genome size, *sensu* Greilhuber 2005) of *A. kamchatica* is the average of the two parental species, in the subset of data including *A. kamchatica*, *A. h. gemifera* and *A. lyrata* (two subspecies were combined). A linear mixed-effects model was fitted with 1Cx values as the dependent variable, population as a random effect and species as a fixed effect, and the linear contrast, (1Cx of *A. kamchatica*) = [(1Cx of *A. h. gemifera*) + (1Cx of *A. lyrata*)]/2, was tested with an R package, *multcomp*. For estimates of genome size diversity in each taxon, we used the CV among populations in 2C DNA content with the bias correction (Sokal and Rohlf 1995). To estimate genome size diversity in diploid *A. thaliana*, we used data from Schmuths *et al.* (2004) collected from 18 worldwide accessions using the same flow cytometry methods that we used.

## Results

**Figure 3.**
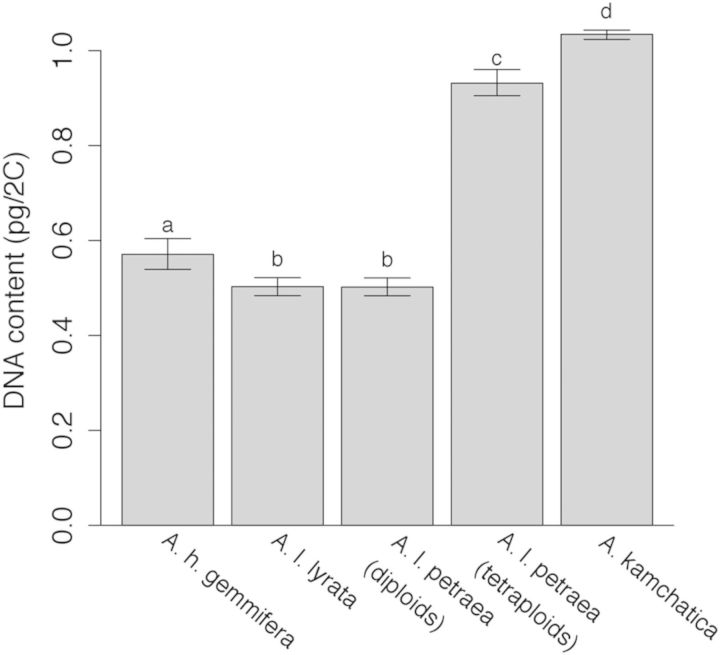
Estimates of 2C DNA content (pg) of each taxon and the 95 % confidence intervals of the estimates. Letters indicate significant differences (*P*< 0.05) based on Tukey's post hoc comparisons.

We found that 2C DNA content in *A. kamchatica* populations varied from 1.013 to 1.083 pg/2C, with a mean 2C DNA content of 1.034 ± 0.005 pg/2C (mean ± SE). *Arabidopsis kamchatica* and two of the *A. lyrata* ssp. *petraea* samples (Austria and Dürn, Germany) had approximately double the genome size of the other *A. lyrata (*ssp. *lyrata and* ssp. *petraea*) and *A. halleri* ssp. *gemmifera* samples (Fig. 3, Table 1). These taxa significantly differed in nuclear DNA content (*D* = 136.18, df = 1, *P* < 0.0002). These results, when taken together with chromosome counts and flow cytometry results conducted by Dart *et al.* (2004) in some of the same collections we used, suggest that the majority of *A. l. lyrata*, *A. l. petraea* and *A. h. gemmifera* are diploids, while *A. kamchatica* and two *A. l. petraea* samples are tetraploids (Fig. 3; Table 1).

There was significant variation in genome size among *A. kamchatica* populations (*F*_8,39_ = 15.7, *P* < 10^−9^). Post hoc tests indicate that the genome size of the Japanese *A. kamchatica* population (Shinbo) was significantly larger than the North American populations. The Canadian *A. kamchatica* population did not differ in genome size from the Alaskan populations. However, two of the six Alaskan populations differed in genome size; the nuclear DNA content of the Goodnews Bay population was 3 % smaller than that of the Grant Lagoon population. Despite the minor amounts of variation among populations, none of the *A. kamchatica* plants sampled appear to be diploid.

The 2C DNA content of *A. l. lyrata* (0.503 pg/2C, 95 % CI [0.484, 0.522]) and diploid *A. l. petraea* (0.502 pg/2C, 95 % CI [0.484, 0.521]) did not significantly differ from one another (Fig. 3). The *A. h. gemmifera* genome (0.571 pg/2C, 95 % CI [0.539, 0.604]) was 14 % larger than *A. l. petraea* and *A. l. lyrata* (Fig. 3). We did not have enough samples/population of these taxa to analyse differences among populations.

**Figure 4.**
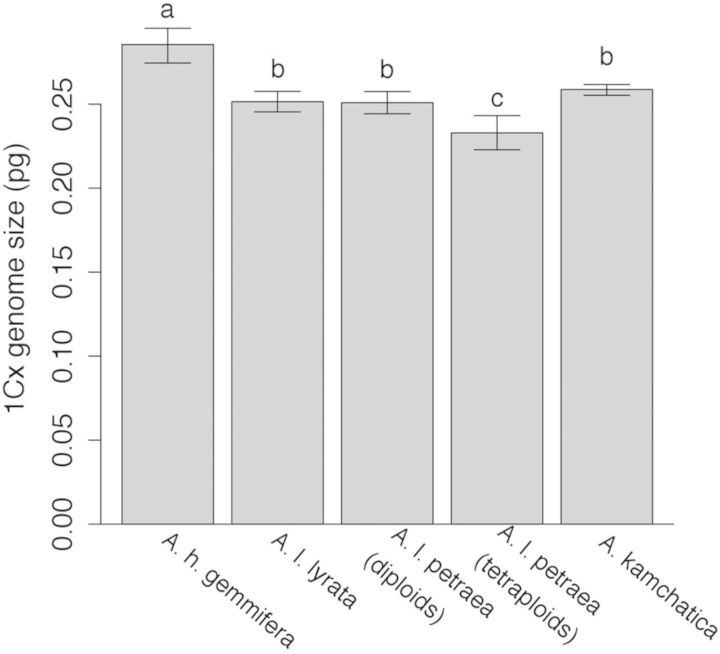
Estimates of 1Cx (haploid) genome size (pg) of each taxon. The error bars are 95 % confidence intervals of the estimates. Letters indicate significant differences (*P* < 0.05) based on Tukey's post hoc comparisons.

*Arabidopsis kamchatica* appears to have been derived through allopolyploidy from *A. lyrata* and *A. h. gemmifera* (Shimizu-Inatsugi *et al.* 2009). Thus, if polyploidization was recent, and there were no subsequent changes in genome size, we would predict that the genome size of the allotetraploid should be equal to the sum of the two parental taxa. Further, the 1Cx genome size (i.e. the haploid genome size, *sensu* Greilhuber 2005) should be an average of its parents. However, *A. kamchatica*, on average, is slightly smaller than expected. Comparing the 1Cx genome sizes of *A. kamchatica* to its parents (Fig. 4), we can see that the *A. kamchatica* 1Cx genome size is intermediate to its parents, but less than the average of its parents (*A. kamchatica*: 0.259 pg; mean of parents: 0.268 pg, *z* = −2.81, *P* = 0.0049). Further, it is not significantly different from the smaller parent, *A. lyrata* (Fig. 4), suggesting that *A. kamchatica* may have lost DNA. *Arabidopsis kamchatica* appears to have lost ∼37.594 Mbp/2C of DNA or 3.6 % of its genome. Autotetraploid *A. l. petraea* also appears to have lost DNA. The mean 1Cx genome size of tetraploid *A. l. petraea* (0.233 pg, 95 % CI [0.223, 0.243]) is less than the 1Cx content of diploid *A. l. petraea* (0.251 pg, 95 % CI [0.244, 0.258]), a loss of ∼70.366 Mbp/2C, or 7.2 % of the genome.

**Figure 5.**
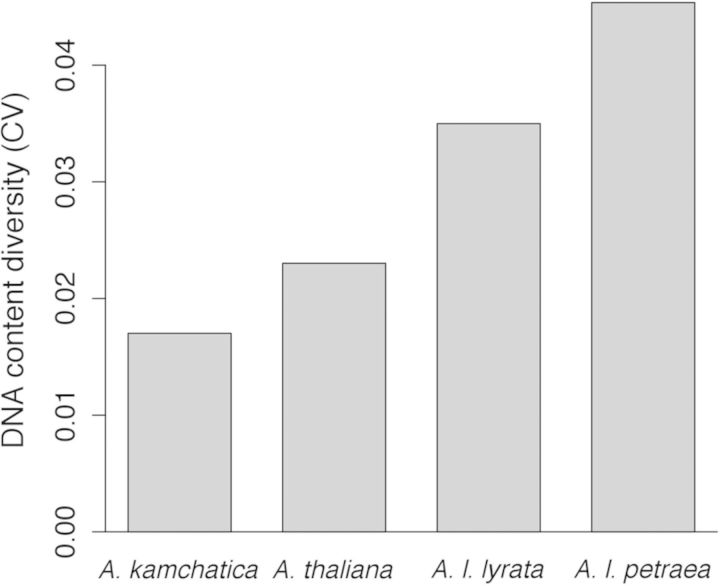
Genome size diversity in *Arabidopsis* taxa, measured as CV in 2C DNA content. Only diploid *A. thaliana* and *A. l. petraea* are included because there were too few tetraploids to estimate CV (two from each taxon).

We were able to estimate genome size diversity (i.e. the CV in 2C DNA content) in *A. kamchatica*, *A. l. petraea*, *A l. lyrata* and *A. thaliana*, which were all sampled from multiple populations (*A. thaliana* data were from Schmuths *et al.* 2004). *Arabidopsis kamchatica* has the lowest diversity of all the *Arabidopsis* taxa studied (Fig. 5), including *A. thaliana* (Schmuths *et al.* 2004).

## Discussion

### Reliability of ploidy estimates

Our genome size estimates are very similar to those of Dart *et al.* (2004) for diploid and tetraploid collections in common (Table 1), suggesting that our results are reliable. Using both chromosome counting and flow cytometry, Dart *et al.* (2004) found that plants from Japan (Shinbo) and Austria are tetraploid (2*n* = 4*x* = 32) with genome sizes of 1.1 pg/2C (Japan) and 0.9 pg/2C (Austria), while plants from Iceland are diploid (2*n* = 2*x* = 16) with a genome size of 0.52 pg/2C. The small differences between our data and those of Dart *et al.* (2004) are likely due to the fact that Dart *et al.* (2004) used fluorescent beads as an internal size standard, whereas we used leaf tissue from *G. max*. While beads are sufficient for ploidy determination, leaf tissue is the preferred internal size standard for absolute genome size estimation because staining variation can be taken into account (Doležel *et al.* 2007).

### No ploidy variation within *A. kamchatica*

Several previous reports have suggested that *A. kamchatica* contains both diploid and tetraploid individuals (Dawe and Murray 1981; Wang *et al.* 2010). While many species show a mix of ploidy levels, even within a population, these are likely autopolyploids (Schmuths *et al.* 2004; Jørgensen *et al.* 2011). Given that *A. kamchatica* is an allopolyploid, diploids spontaneously produced from tetraploids would likely have low vigour and fertility (Kerber 1964; Ladizinsky and Fainstein 1978), as allopolyploidization appears to rapidly result in gene silencing and gene loss for numerous loci (Kashkush *et al.* 2002; Adams and Wendel 2005). Our data from 52 *A. kamchatica* specimens representing most of the species' range found no evidence of diploid *A. kamchatica*, and we suggest that the species is likely to be entirely tetraploid. If diploids are present, they are likely to be in very low frequencies, and not maintained by selection.

Deeper investigation into previous reports also suggests that there is no good evidence for the presence of diploid *A. kamchatica*. Dawe and Murray (1981) report chromosome counts from three diploid (2*n* = 2*x* = 16) and two tetraploid *A. kamchatica* samples (2*n* = 4*x* = 32). *Arabidopsis kamchatica* is very difficult to morphologically distinguish from mostly diploid *A. lyrata*; however, molecular data suggest that the two species have distinct geographical ranges (Schmickl *et al.* 2010). The tetraploid counts reported by Dawe and Murray (1981) are within the species range of *A. kamchatica* suggested by Schmickl *et al.* (2010), whereas two of the three diploid counts are from plants growing north of the Brooks Range in Alaska and are probably *A. l. petraea* (Schmickl *et al.* 2010) or *A. media* (Mulligan 1995). One of the diploid counts (originally reported in Dawe and Murray 1979) comes from well within *A. kamchatica*'s range in interior Alaska, near several of our collections (63°02′N, 145°29′W), and was likely taken from *A. kamchatica*. However, Mulligan (1995) claims that the diploid report is an error, and that the voucher in ALA indicates that 2*n* = 32 (tetraploid), not 2*n* = 16 (diploid). Other chromosome counts reported for *A. kamchatica* by Mulligan (1995) are all tetraploid, and he suggests that the species is entirely tetraploid.

Wang *et al.* (2010) claim to have detected both diploid and tetraploid *A. kamchatica* in Taiwan using flow cytometry and sequencing of nuclear DNA from 98 genes. They suggest that diploids have a ‘mosaic genome’ of the two parental species. Although this would be very interesting if confirmed, more complete evidence is desirable. First, their flow cytometry runs seem to lack an internal standard. The absolute value of nucleus fluorescence cannot reliably be used to estimate genome size as this value shifts due to variation in sample preparation, staining and analysis (Doležel *et al.* 2007). This shift can be seen by comparing Fig. S1A and S1B in Wang *et al* (2010), which were presented as evidence of diploid and tetraploid *A. kamchatica*. Further, their DNA sequence data do not provide any evidence of ploidy since only a single clone per PCR reaction was sequenced, ensuring that only a single homeologue (randomly chosen from one of the two parental genomes of tetraploids) could be obtained from each individual (Wang *et al.* 2010). Although we have not sampled *A. kamchatica* from Taiwan for our study, the ‘mosaic genome’ of purported diploid *A. kamchatica* can possibly be explained by misinterpretation of flow cytometry data and randomly sequencing only one of the two homeologues from each gene.

### DNA content variation within *A. kamchatica*

We appear to have identified variation in the 2C DNA content among *A. kamchatica* populations. Greilhuber (2005) suggested that a great deal of apparent within-species, within-ploidy variation in genome DNA content estimated by flow cytometry is due to methodological artefacts. For instance, different levels of anthocyanins, tannic acid and other secondary metabolites in leaves can influence fluorescence and apparent DNA content (Loureiro *et al.* 2006; Bennett *et al.* 2008). Following best-practice recommended protocols (Doležel *et al.* 2007), we used an internal size standard co-chopped with each sample, we used Otto's buffer, which reduces the effects of tannic acid (Loureiro *et al.* 2006), and leaves were not pigmented. Further, repeated measurements of the same plant on different days produced very similar DNA content estimates (<1.1 % variation). Thus the variation we observed should be biologically real (Schmuths *et al.* 2004). However, co-chopping two putatively different samples from different populations would further increase certainty that differences among populations are not artefactual (Greilhuber 2005).

The 2C DNA content of Japanese *A. kamchatica* appears to be slightly larger than North American *A. kamchatica*. This observed genome size difference may differentiate the two *A. kamchatica* subspecies: *A. kamchatica* ssp. *kamchatica* and *A. kamchatica* ssp. *kawasakiana*. Our Japanese *A. kamchatica* samples are from subspecies *A. k. kawasakiana*, whereas the rest of our samples represent subspecies *A. k. kamchatica* from North America. These two subspecies differ in habitat, morphology and nucleotide allele frequencies (Shimizu-Inatsugi *et al.* 2009; Higashi *et al.* 2012), and Shimizu-Inatsugi *et al.* (2009) suggested that *A. k. kawasakiana* may represent a distinct origin of *A. kamchatica*. The difference in genome size between the Japanese *A. k. kawasakiana* and North American *A. k. kamchatica* potentially supports that hypothesis. Alternatively, ongoing hybridization between *A. kamchatica* and its diploid parent, *A. h. gemmifera*, in Asia (Wang *et al.* 2010) could increase the genome size in Asia by reintroducing homeologues that may have been deleted in the allotetraploid.

Other possible explanations for the genome size differences between Japan and North America include biogeographic history and selection. It has been suggested that time-limited environments may select for a smaller genome with more rapid cell division (reviewed in Šmarda and Bureš 2010). As *A. kamchatica* expanded north out of Japan and across the cold Bering land bridge into North America (Shimizu-Inatsugi *et al.* 2009), a smaller genome may have been favoured due to the short growing season. Interestingly, despite the difference in genome size, Japanese and North American samples appear to have lost similar numbers of genes (P. L. Chang, unpubl. res.). Our sampling from Japan was very limited. A thorough investigation of genome size variation from throughout Japan, accompanied by an investigation of introgression and deletions, is needed for a thorough understanding of genome size evolution in this species.

Within-species variation in nuclear genome size may be an important source of genetic diversity, especially if it is associated with phenotypic and ecological variation (Levin 2002; Matsushita *et al.* 2012). Although we did find significant levels of genome size diversity in the allotetraploid *A. kamchatica*, levels of genome size diversity were much lower than in the diploid *Arabidopsis* taxa studied (Fig. 5). This is consistent with the low levels of nucleotide diversity in *A. kamchatica* relative to the other taxa studied (Shimizu-Inatsugi *et al.* 2009). Although nucleotide diversity is generated by point mutations, while genome size variation is generated by indels, changes in repetitive DNA and transposon activity (Šmarda and Bureš 2010; Long *et al.* 2013), the two forms of genetic diversity are likely to be governed by many of the same population genetic processes such as mating system, biogeography and demographic history (Loveless and Hamrick 1984; Ingvarsson 2002; Glémin *et al.* 2006; Duchoslav *et al.* 2013).

### Loss of DNA in tetraploids

The DNA content of tetraploid *A. kamchatica* was slightly less than expected based on the sum of the two parental taxa. It is possible that this apparent loss in DNA content could be artefactual, due to differences between species in plant secondary compounds (Greilhuber 2005). However, rapid loss of DNA after polyploidization appears to be common in polyploids, as the 1Cx genome size has been shown to decrease as the ploidy level increases (Bennett and Thomas 1991; Raina *et al.* 1994; Ozkan *et al.* 2001; Leitch and Bennett 2004; Angulo and Dematteis 2013; Duchoslav *et al.* 2013). Bennett and Thomas (1991) suggest that these changes in DNA content may have adaptive significance, perhaps because the rate of cell division is slowed considerably as genome size increases (Bennett 1972) and it may be beneficial to remove unnecessary DNA when ploidy level is high.

The majority of genome size variation within plant species at a single ploidy level is due to variation in amounts of repetitive DNA such as transposable elements, ribosomal genes and centromeric repeats (Levin 1993; Davison *et al.* 2007; Šmarda and Bureš 2010; Long *et al.* 2013). However, polyploids may also lose considerable amounts of functional DNA either because it is not necessary to have two copies or because it may allow the two parental genomes to resolve incompatibilities (Kashkush *et al.* 2002; Adams and Wendel 2005; Buggs *et al.* 2012). Whole-genome sequencing of *A. kamchatica*, and comparison to its parental taxa, suggests that each of three accessions from different geographic regions lost ∼463 of more than 60 000 total genes (∼2 % of assembled genes; P. L. Chang, unpubl. res.). Considering that our flow cytometry estimate of the *A. kamchatica* genome size was 3.6 % smaller than expected based on the sum of the parental genomes, the total amount of DNA lost is comparable to the percent of genes lost. This suggests that DNA was lost from both genic regions and non-functional regions in *A. kamchatica*.

*Arabidopsis l. petraea* tetraploids appear to have lost considerably more DNA than *A. kamchatica*. Although these plants are thought to be *A. l. petraea* autotetraploids, they may have experienced hybridization and introgression of DNA from *A. arenosa* (Jørgensen *et al.* 2011; Schmickl and Koch 2011), which has a genome size that is 13 % smaller than *A. l. petraea* (Jørgensen *et al.* 2011). Using DNA content numbers from Jørgensen *et al.* (2011), *A. l. petraea* tetraploid genomes are just slightly smaller than expected from the sum of diploid *A. l. petraea* and diploid *A. arenosa* genomes: observed tetraploid *A. l. petraea* relative genome size 0.44; vs expected diploid *A. l. petraea* 0.23 + diploid *A. arenosa* 0.20 = 0.43 (data are presented as a ratio of the sample peak over the internal standard peak, and cannot be converted to picograms since the 2C DNA content of the standard, *Ilex crenata*, is unknown; Jørgensen *et al.* 2011). The apparent loss of DNA in tetraploid *A. l. petraea* may thus be largely due to hybridization rather than gradual DNA loss through diploidization.

## Conclusions

Contrary to some prior reports, all *A. kamchatica* plants in our samples appear to be tetraploid. We found that the allotetraploid, *A. kamchatica*, has a genome size that is just slightly less than the sum of its diploid parental taxa, *A. l. petraea* and *A. h. gemmifera*. Genome size diversity was lower in *A. kamchatica* than in other *Arabidopsis* taxa. However, there was some variation in genome size, where North American populations of *A. k. kamchatica* seem to have lost slightly more DNA than the Japanese population of subspecies *A. k. kawasakiana*. The development of *A. kamchatica* into a model system for the study of polyploidy has the potential to yield a great deal of insight, as its parental taxa have been well studied at both the ecological and genetic levels, and myriad molecular tools from *A. thaliana* are available.

## Sources of Funding

This research was supported by the National Center for Research Resources (NCRR) at the National Institutes of Health (NIH) (Alaska INBRE) [RR016466 and 5P20RR016466] and the National Science Foundation (NSF) Experimental Program to Stimulate Competitive Research (Alaska EPSCoR) [0346770].

## Contributions by the Authors

Plants were collected by J.A.S., D.E.W. and N.T. Methods were developed and debugged by J.A.S., G.J.H. and N.T. J.A.S. collected the data. J.A.S. and N.T. analysed the data and produced figures. D.E.W. conceived and wrote the manuscript, which was edited by J.A.S., G.J.H. and N.T.

## Conflicts of Interest Statement

None declared.
